# Amyloid-beta peptide (25–35) triggers a reorganization of lipid membranes driven by temperature changes

**DOI:** 10.1038/s41598-021-01347-7

**Published:** 2021-11-09

**Authors:** Oleksandr Ivankov, Tatiana N. Murugova, Elena V. Ermakova, Tomáš Kondela, Dina R. Badreeva, Pavol Hrubovčák, Dmitry Soloviov, Alexey Tsarenko, Andrey Rogachev, Alexander I. Kuklin, Norbert Kučerka

**Affiliations:** 1 Frank Laboratory of Neutron Physics, Joint Institute for Nuclear Research, 141980 Dubna, Russia; 2 Institute for Safety Problems of Nuclear Power Plants, NAS of Ukraine, 03028 Kyiv, Ukraine; 3 Department of Nuclear Physics and Biophysics, Comenius University Bratislava, 842 48 Bratislava, Slovakia; 4 Laboratory of Information Technologies, Joint Institute for Nuclear Research, 141980 Dubna, Russia; 5 Department of Condensed Matter Physics, University of P. J. Šafárik in Košice, 041 54 Košice, Slovakia; 6 Moscow Institute of Physics and Technology, 141701 Dolgoprudny, Russia; 7 Department of Physical Chemistry of Drugs, Faculty of Pharmacy, Comenius University Bratislava, 832 32 Bratislava, Slovakia

**Keywords:** Membrane biophysics, Membrane structure and assembly, Biomaterials - proteins, Atomistic models, Biological physics

## Abstract

The amyloid-beta peptide (Aβ) is considered a key factor in Alzheimer's disease (AD) ever since the discovery of the disease. The understanding of its damaging influence has however shifted recently from large fibrils observed in the inter-cellular environment to the small oligomers interacting with a cell membrane. We studied the effect of temperature on the latter interactions by evaluating the structural characteristics of zwitterionic phosphatidylcholine (PC) membranes with incorporated Aβ_25–35_ peptide. By means of small angle neutron scattering (SANS), we have observed for the first time a spontaneous reformation of extruded unilamellar vesicles (EULVs) to discoidal bicelle-like structures (BLSs) and small unilamellar vesicles (SULVs). These changes in the membrane self-organization happen during the thermodynamic phase transitions of lipids and only in the presence of the peptide. We interpret the dramatic changes in the membrane's overall shape with parallel changes in its thickness as the Aβ_25–35_ triggered membrane damage and a consequent reorganization of its structure. The suggested process is consistent with an action of separate peptides or small size peptide oligomers rather than the result of large Aβ fibrils.

## Introduction

One of the hallmarks of Alzheimer's disease (AD) is a self-aggregation of the amyloid-β (Aβ) peptide into the large extracellular β-sheet fibrils^1,2^. Aβ peptide is known to be cleaved enzymatically from a transmembrane amyloid precursor protein (APP) in 1–40 and 1–42 isoforms. The transformation from the membrane monomeric peptide to an aggregated fibril outside the membrane, however, has yet to be understood. It was shown nevertheless, that these peptides and their interactions with the membrane in particular, may play a major role in triggering the onset of AD^3^.

The changes in membrane functionality most likely originate in the structural properties of the membrane. For example, the thermodynamic phase of lipids plays one of the starring roles in determining the membrane’s structural properties^4^. At the assistance of increasing temperature, the crystalline phase passes from a highly ordered structure to the liquid-crystalline phase typical of the high disorder. The ability of the Aβ to interact with the membrane was found to depend on the structural and elasto-mechanical properties of the membrane, thus its thermodynamic phase^5,6^. An intriguing possibility would be to look for a membrane state that will retain the peptides in their monomeric form.

There are three canonical mechanisms of action proposed for the membrane disruption by the Aβ: interaction of fibrils with the membrane, pore formation, and membrane damage by monomeric Aβ or their small oligomers (detergent-like interactions)^7–9^. According to the third model proposed, the cytotoxic effect is viable through a mechanism of lipid extraction by these aggregates. The size of aggregates observed usually depends on the peptide to lipid ratio^10^. The amyloidogenic aggregation typically happens at high ratios, while it would be an irreversible process supposedly corresponding to the onset of the disease. Our study on the other hand focuses on pre-clinical AD stages where Aβ appears in its non-aggregated forms, where we scrutinize the peptide-membrane interactions instead of those between the peptides themselves. The latter has been observed ample times and can be accelerated significantly when the temperature increases. Accordingly, we take care to incorporate monomeric Aβ at very low concentrations into the membrane during its formation rather than adding it afterwards^5,11–13^. After initial preparation and stabilization, our model membrane systems do not appear to be affected dramatically by the accommodation of Aβ, which is consistent with its homogeneous distribution. Further, our final results advocate the interactions of a major part of vesicles in our samples with many separate peptides during the membrane phase transition. This was indeed proposed previously^14^.

Despite a long-lasting debate on the character of the lipid membrane phase transition, the changes of physico-chemical properties of the membrane do not result in its disruption. The interaction with its additional components may nevertheless introduce some abrupt structural changes^15,16^. Our report documents such changes triggered by the presence of Aβ_25–35_ during the phase transitions of the underlying lipid matrix. The observed effect of Aβ peptide fragmenting the otherwise cohesive membrane may be a key point in understanding the role of membrane–peptide interactions in AD, all the more we observe it in the case of a simple model system.

The Aβ_25–35_ was chosen to mimic the smallest transmembrane part of the Aβ peptide while still including its toxic fragment. The studies of the conformational changes of Aβ_25–35_ and Aβ_1–42_ in the presence of the lipid membrane show little difference in the behavior regarding their incorporation in the membrane^17^, influence on its fluidity^6^ and other mechanical properties as well as the toxicity^18,19^. Further, in the present study, we aim to find the structural characteristics of the non-electrostatic peptide-membrane interactions connected to the temperature and/or thermodynamic phase of the model membrane shown by Yoda et al.^5^. Accordingly, the membrane is modeled purely by the zwitterionic lipids. Our results confirm a strong interplay between the chosen Aβ fragment and model membrane, and show an intriguing behavior even at this model level.

The neuronal cell membrane is modeled by one of its main zwitterionic components, namely phosphatidylcholine (PC), with either 16:0 or 14:0 chains that are also ones of the most represented lipidomic elements in the human brain^20^. Although the most physiological combination of hydrocarbon chains appears to be that of 16:0 and 18:1, constructing the membrane of DPPC (i.e., diC16:0PC) or DMPC (i.e., diC14:0PC) allowed us to regulate the phase transition temperature in a physiological range and examine its effect purposely. Their pretransition and main phase transition temperatures are 34.2 and 41.4 °C in the case of DPPC, and 14.3 and 23.9 °C in the case of DMPC, respectively^21^.

## Results and discussion

**Figure 1:**
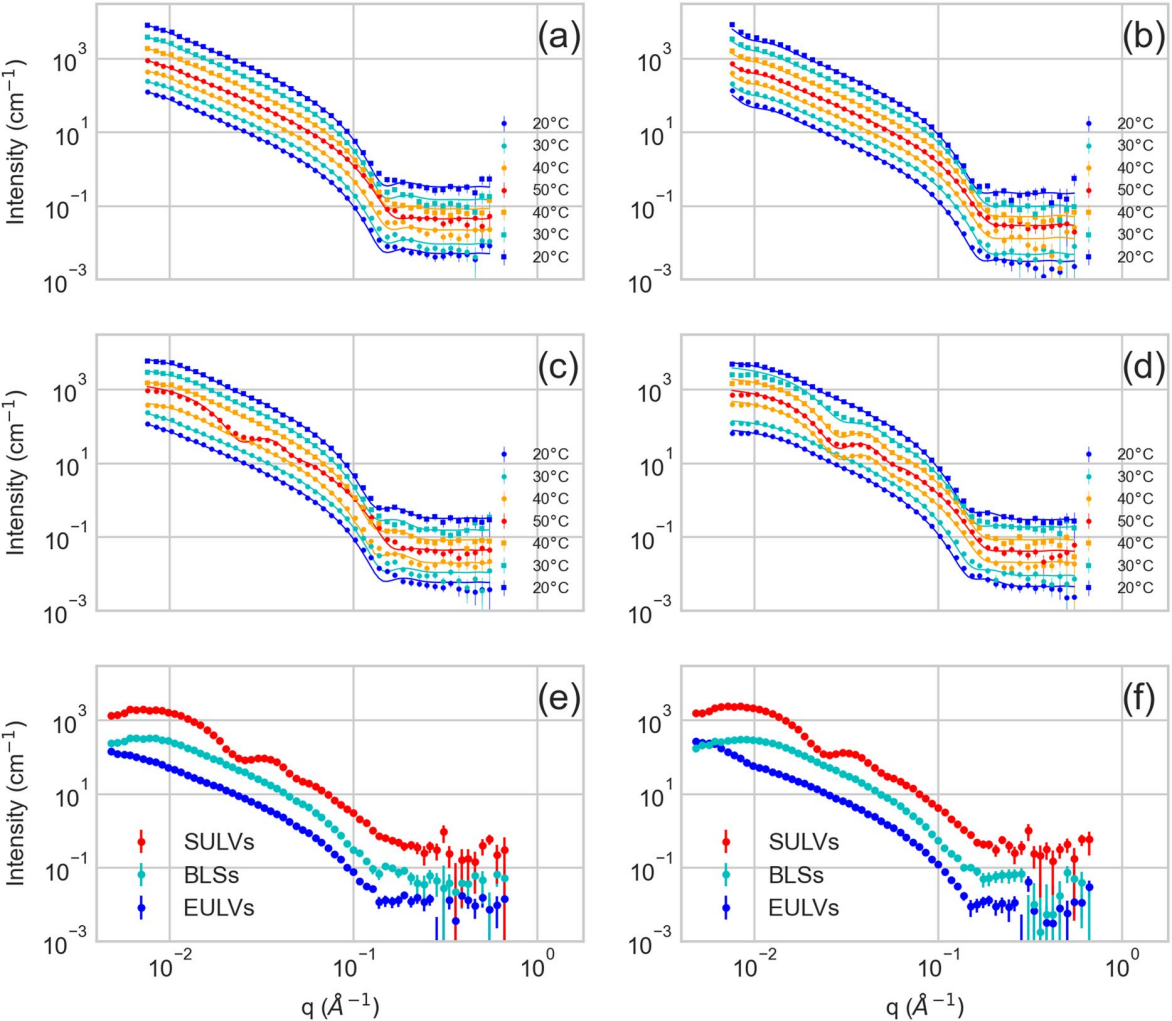
SANS curves for the DPPC (**a**), DPPC/Aβ_25–35_ (**c**), DMPC (**b**), and DMPC/Aβ_25–35_ (**d**) unilamellar vesicles prepared via the extrusion procedure and collected at various temperatures depicted in legends. Note a mutual multiplication factor of 2 utilized to shift the data for their better visualization. The commensurately colored solid lines correspond to the best fits according to an appropriate model approximation as described in the text. The low-q enhanced data for DPPC/Aβ_25–35_ (**e**) and DMPC/Aβ_25–35_ (**f**) emphasize the differences in the overall shape of membrane organization (SULVs vs. BLSs vs. EULVs).

The lipid membranes with Aβ peptide incorporated initially were forced to form ULVs of well-defined sizes and bilayer thicknesses by the extrusion through 500 Å pores. The diluted suspension of EULVs was utilized as the best model for structural investigations by SANS that offers the desired structural parameters. The SANS curves were collected at various temperatures below and above the mentioned phase transitions for a control system of neat DPPC or DMPC system (DPPC or DMPC, respectively) and systems of the Aβ_25–35_ loaded DPPC or DMPC bilayers (DPPC/Aβ_25–35_ or DMPC/Aβ_25–35_, respectively) as shown in Fig. 1a,b and c,d, respectively. While the SANS curves for the neat lipid systems (Fig. 1a,b) at various temperatures display differences exclusively in a high-q region (above 0.1 Å^−1^), those collected for the peptide included systems (Fig. 1c,d) display significant changes also in a low-q region (below 0.02 Å^−1^). This is emphasized further by the data obtained in a separate experiment that was optimized for the low-q region (Fig. 1e,f; see also Fig. S1). Similar changes in the SANS curves were observed for some systems under certain conditions previously and were connected to the formation of bicelles^15,16,22,23^. By virtue of the scattering experiment principles, the low-q region of presented data is influenced for the most part by a form factor of the overall ULV, while the high-q region is influenced by a form factor of the bilayer, and its thickness in particular. Based on the changes observed in our SANS curves, we can thus evaluate the temperature induced variations in the membrane thickness, and ULVs’ size and shape.

The membrane thicknesses were obtained at first from the small-angle Kratky–Porod approximation for unoriented lamellar objects^24^. This approach is very sensitive to a possible Bragg-scattering peak that would skew the data analysis. The lack of its detection, however, allows us to conclude the absence of long-range order in our systems. On the other hand, this approach is not suitable for extracting further information regarding the object shape and size, which prompted us to employ a 3-shell vesicle model^25^. It is worth noting nevertheless, that the changes in the obtained membrane thicknesses were found independent of the approximation model used (see Fig. S2 of the supplement).

**Figure 2:**
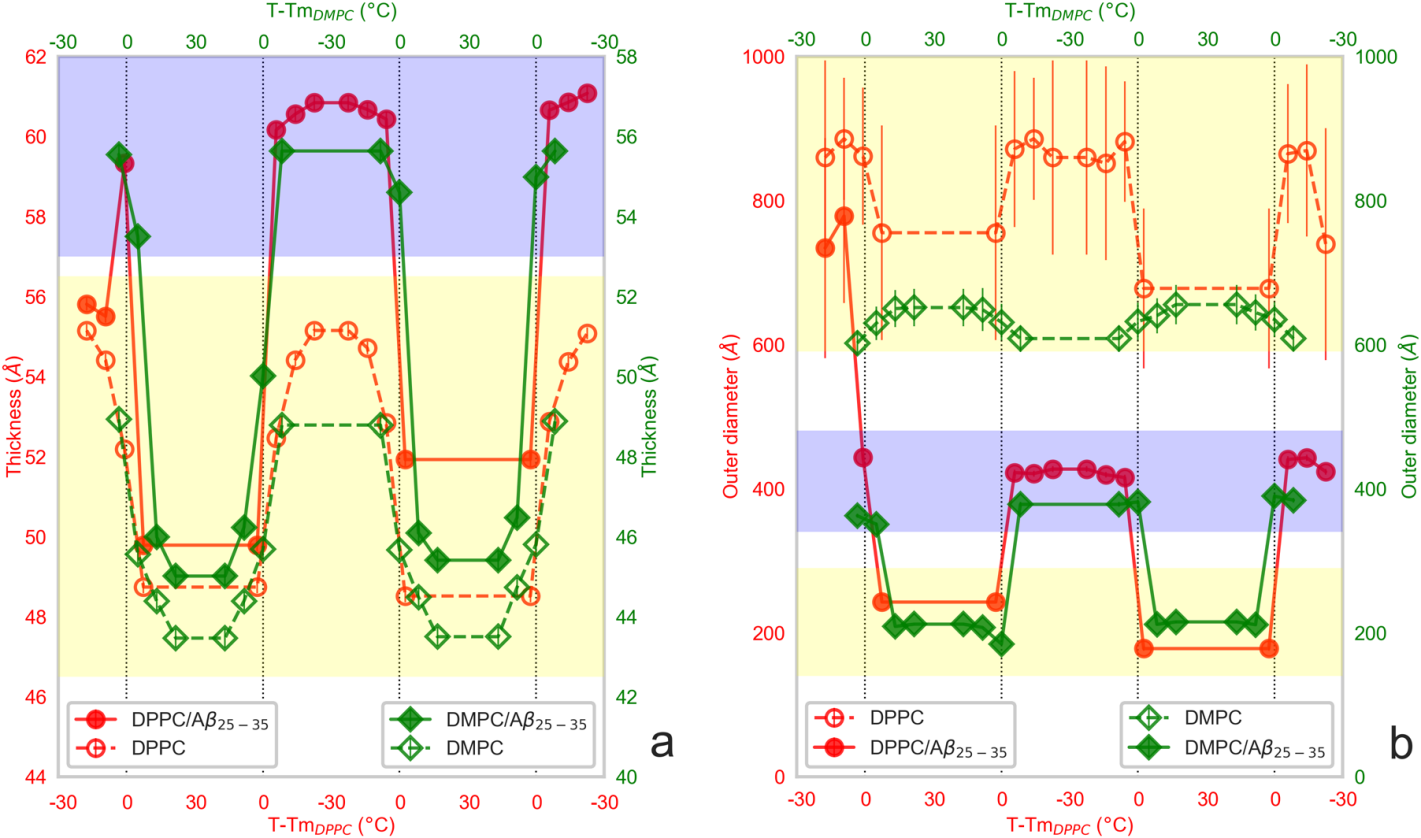
The membrane thicknesses (**a**) and outer diameter of lipid aggregates (**b**) obtained for the systems based on DPPC (red color; left and bottom axes) or DMPC (green color; right and top axes) as a function of reduced temperature relative to main transition (Tm_DPPC_ = 41 °C and Tm_DMPC_ = 24 °C). The order of points from left to right corresponds to the chronology of SANS measurements. The neat lipid systems are shown by open symbols and those with the addition of Aβ_25–35_ by solid ones. The vertical lines mark the phase transitions for neat lipid systems as reported in the literature. The horizontal shaded areas demarcate the structural forms of membranes by yellow color in the case of ULVs (EULVs and/or SULVs), and by blue color in the case of BLSs.

The changes to the bilayer thickness are shown in Fig. S3 of the supplement. Note that the measurements at the same temperatures result in a different number of points in different thermodynamic phases for the two lipid systems (i.e., there is 1 point below and 3 points above the main phase transition in the case of DMPC bilayers, while 3 points below and 1 point above the main phase transition in the case of DPPC bilayers). We thus display these results as a function of temperature reduced relative to the main transition of a given lipid in Fig. 2. This representation makes it apparent, that the behavior of bilayer thickness for the two systems is similar when the comparison is done within the same phase, only shifted by ~ 4 Å due to differences in hydrocarbon chain lengths^26^. As expected, the bilayer thickness decreases within highly ordered gel (L_β_) and/or ripple (P_β′_) phases gradually at first due to a continuously increasing disorder of hydrocarbon chains^27,28^. A sudden change in the bilayer thickness is then observed upon DMPC and DPPC transitioning from P_β′_ to liquid-crystalline (L_α_) phase^27^. Finally, the thickness increases during the reverse transition. It is worth noting, the thickness variations due to these temperature changes are fully reversible in the case of neat lipid systems (empty symbols in Fig. 2a).

Intriguingly, a different behavior was observed in the case of Aβ_25–35_ loaded bilayers. At first, DPPC/Aβ_25–35_ bilayer thickness appears to increase minimally by the incorporation of Aβ (see the solid red circles at the initial two points in Fig. 2a). During the heating, however, the thickness shows a startling increase over the temperature interval where the neat DPPC bilayer undergoes its L_β_–P_β′_ pretransition (T − T_mDPPC_ = − 7 °C)^21^. It is of the utmost interest to note, that in contrast to the DPPC system, the heating process brought up the changes not only to the bilayer thickness but also to the overall shape of ULVs as can be detected already in Fig. 1b (low-q region). The SANS curves collected at T = 40 °C could be fitted with the bilayered vesicle model poorly, while the satisfactory results were obtained by employing a model of randomly oriented cylindrical shells with a circular cross-section (bicelle-like structures—BLSs)^29^. The dramatic changes to the membrane thickness were, however, independent of the model describing an overall shape of the membrane (see Fig. S2 of the supplement). The initially produced EULVs of approximately 800 Å outer diameter appear to transition to BLSs with their outer diameter of about 400 Å upon heating the DPPC/Aβ_25–35_ to 40 °C as shown in Fig. S4a of the supplement, and in Fig. 2b (T − T_mDPPC_ = − 1 °C, solid red circles). This is in a stark contrast to the DPPC system that only forms ULVs with their sizes varying by less than 200 Å over the entire range of temperatures studied.

It is interesting to note that a similar transition was not observed in the case of DMPC/Aβ_25–35_. In fact, Fig. 2 together with Fig. S4b suggest this system occurring in the form of BLSs from the very first measurement. We argue this being a result of the extrusion procedure performed at room temperature, which likely matches or even exceeds the system main phase transition. We have confirmed our conclusion by repeating the initial measurement with a separately prepared sample. The care was taken to prepare the sample at temperatures below the main phase transition of DMPC. The system then indeed formed the EULVs initially (Fig. 1f), before transitioning between BLSs and SULVs with further temperature changes as observed previously. The conclusion that our original DMPC/Aβ_25–35_ system forms BLSs already at our initial measurements is corroborated also by the bilayer being ~ 7 Å thicker than that made of DMPC (solid and empty diamonds on Fig. 2a) and the outer diameter of lipid aggregates being ~ 400 Å (Fig. 2b)—similar to the parameters of BLSs in the case of DPPC/Aβ_25–35_. This observation then suggests the lipid phase transition as a universal feature driving the membrane reorganization triggered by the presence of Aβ_25–35_.

**Figure 3:**
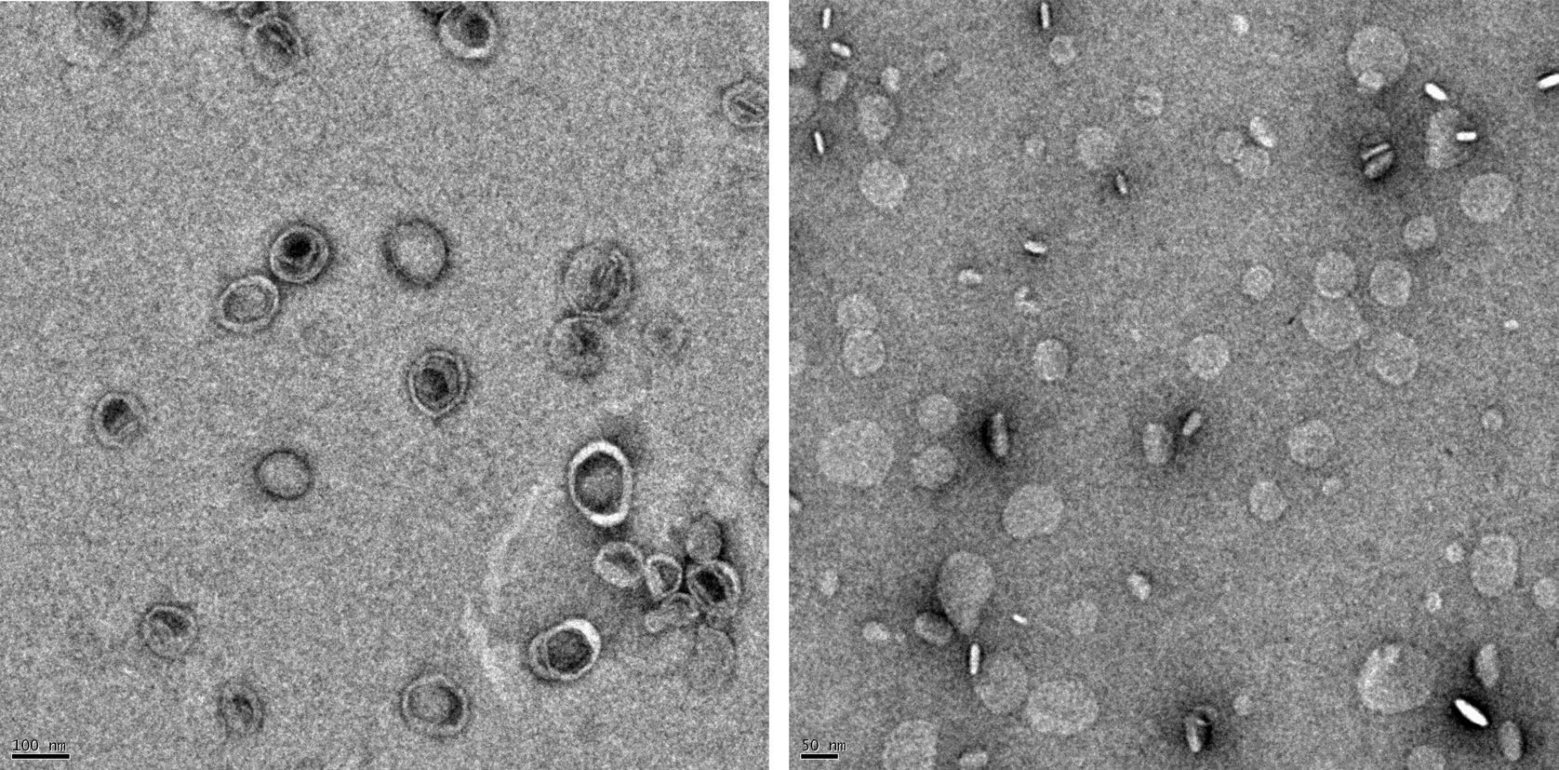
TEM images of the DMPC (left) and DMPC/Aβ_25–35_ (right) systems collected post-SANS measurements at 20 °C. The dark bars (100 and 50 nm, respectively) in the lower-left corners allow assessing of the length scales. Objects in the left-hand panel match the typical 2D projection of vesicular objects with mostly unilamellar walls. The right-hand panel reveals projections of randomly oriented discs also consisting of single layers.

We support our claims of observing the BLSs in the SANS results by observations employing transmission electron microscopy (TEM). The TEM images collected from the samples after their examinations by SANS are presented in Fig. 3. They confirm the structure picture revealed by SANS, showing the objects consistent with the spherical bilayered vesicles in the case of neat DMPC bilayers, and flat discoidal objects when Aβ_25–35_ was incorporated. The DMPC vesicle sizes vary around 900 Å, and the sizes of disc-like structures are around 500 Å, which are in a good agreement with our SANS results. We remind that the two samples were selected for TEM measurements deliberately to confirm the shape of objects assembled during SANS measurements, as our equipment did not allow us to follow the morphological changes while varying temperature. Note also, that the TEM sample preparation procedure may provide slightly different parameters because of additional steps (e.g., dilution, negative staining) employed in the sample preparation.

The formation of BLSs has been proposed as a precursor to the formation of SULVs with low polydispersity^30^. Indeed, our SANS curves for DPPC/Aβ_25–35_ and DMPC/Aβ_25–35_ systems could be fitted satisfactorily with the bilayered vesicle model again after heating the system above the main transition from P_β′_ to L_α_ phase of neat lipid bilayers^21^. The oscillations in SANS curves (Fig. 1c–f, red curves) are born by the narrow distribution of the vesicles’ overall shape and size. Further, the position of the first minimum corresponds inversely to their mean size, allowing the conclusion of them being the low polydispersity SULVs. Our best fit results demonstrate a narrow distribution of ULV outer diameters around a mean value as small as 200 Å (Fig. 2b). At the same time, the bilayer thickness of such SULVs decreases rapidly (Fig. 2a).

**Figure 4:**
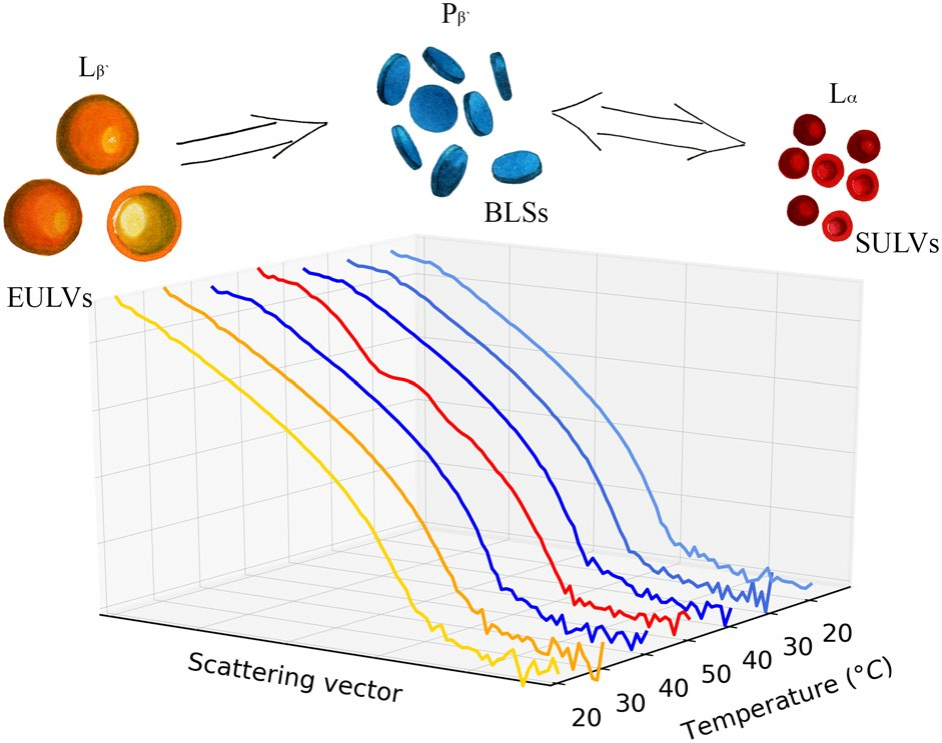
The proposed scheme of the SANS suggested evolution of the DPPC/Aβ_25–35_ membrane organizations during the temperature changes. The SANS curves are colored corresponding to their best fit models shown above. The transition from the initial EULVs to BLSs is irreversible, while the BLSs transition to SULVs reversibly.

Upon cooling the system down again, and further cycling of temperatures between 20 and 50 °C, the corresponding SANS curves corroborate a scenario of the thick-bilayer BLSs at low temperatures and thin-bilayer SULVs at T = 50 °C. We note that the very same samples were examined also utilizing some complementary small angle X-ray scattering measurements, whose results suggested the same behavior (Fig. S5 of the supplement). It is also worth noting that the system has not reached the initial form of EULVs anymore, while it remained to transition between the BLSs and SULVs (see Fig. 4).

Yoda et al.^5^ suggested that the Aβ binds to lipid membranes predominantly in the L_β_ and P_β′_ phase and does not interact with them in the L_α_ phase. Our experimental results show the first and irreversible changes to the DPPC/Aβ_25–35_ membranes over the temperature range corresponding to the transition of neat DPPC bilayers from L_β_ to P_β′_ phase. It suggests then that these changes are related to the thermodynamic properties of the membrane itself. We have performed some MD simulations with the DPPC/Aβ_25–35_ system at different temperatures in an attempt to shed light on these interactions. Simulation results confirm the bilayer thickness increase due to the addition of the peptide and its decrease upon heating the system (Fig. S6 of the supplement). This then indeed supports an essential role of the thermodynamic properties of the membrane through regulating its thickness.

**Figure 5:**
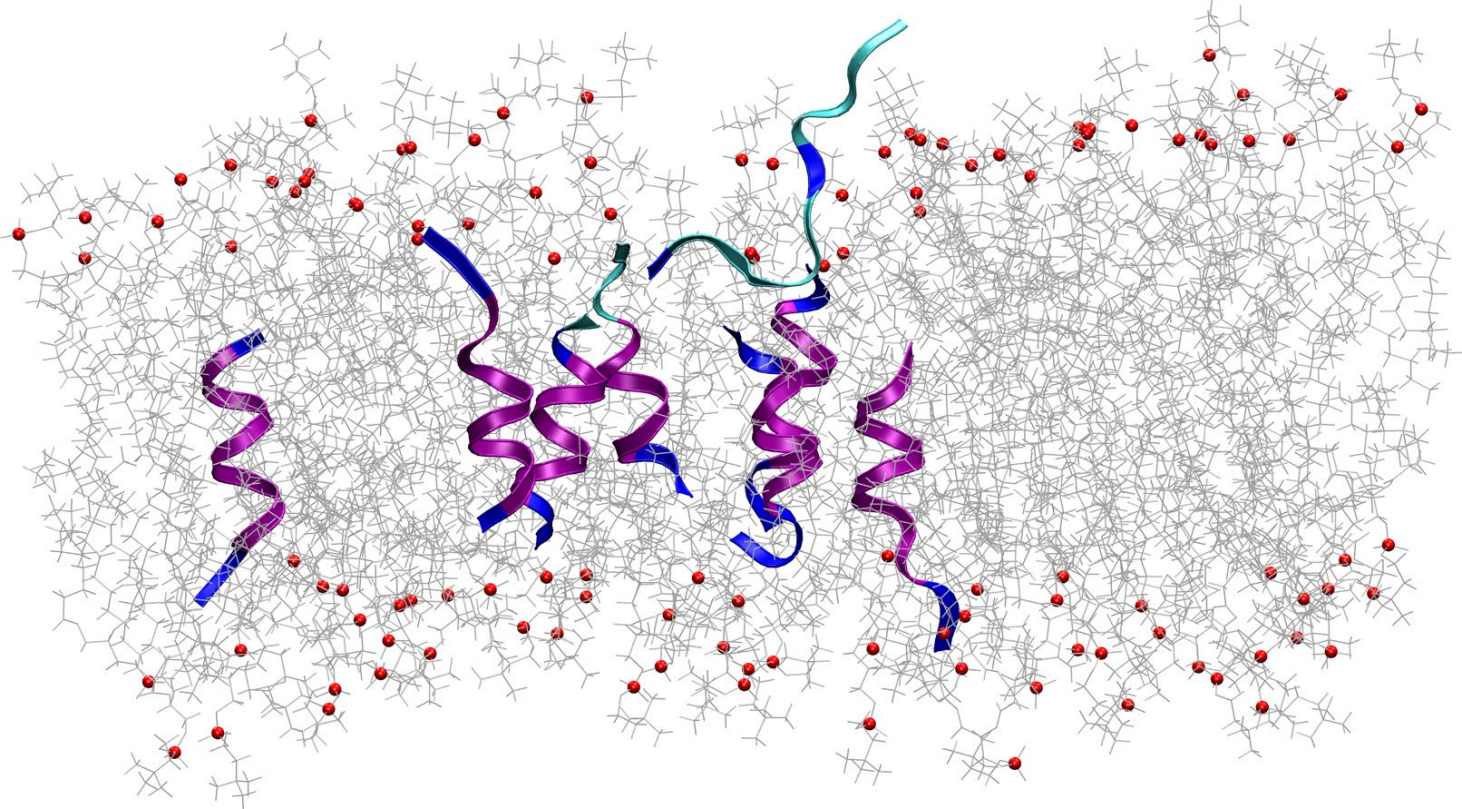
The MD simulations snapshot of the DPPC/Aβ_25–35_ system after its equilibration. The peptides were initially distributed randomly within the membrane plane while oriented parallel to the lipid chains.

The rapid increase of the bilayer thickness accompanied by the change of membrane organization, or a significant shift in the initial location of the peptides was however absent. This could be explained by the limitations of our full-atom simulations that examined a small patch of the membrane only, while some coarse-grain simulations must be employed to access the large-scale aggregations^15^. Our simulations nevertheless reconfirm the plausibility of Aβ_25–35_ incorporation to the DPPC membrane (Fig. 5).

One of the important membrane properties that changes during the phase transition is a lateral diffusion. The Aβ_25–35_ and Aβ_22–40_ have been shown to increase the lateral diffusion in liquid crystalline membranes^31^, and Aβ_22–40_ was suggested to decrease it in membranes near their main phase transition^14^. In addition, the Aβ secondary structure transforms from the α-helix to irregular at the main phase transition^5^. The structural and dynamic changes to the membranes during the phase transition between the less diffusive, while more rigid membrane accompanied also with a relatively rigid α-helix peptide, and the more diffusive fluid membrane with a flexible irregular peptide may be a foundation to a temporary membrane breakage. It is intriguing then to speculate the less flexible α-helix peptide at low temperatures driving a membrane reformation into the BLSs rimmed with the Aβ. This assumption was indeed proposed recently^12^. Such a scenario is consistent also with the Aβ-membrane interactions reported at low temperatures^5^. On the other hand, the increased flexibility of both the membrane and Aβ due to an increased temperature may be a reason for the membrane to reform into the SULVs with canonically distributed Aβ, as we have observed at high temperatures in agreement with findings in the literature^32^.

## Conclusions

Our SANS experiments performed at temperatures ensuing the variation of membrane thermodynamic properties suggest for the first time dramatic changes to the structure of membrane organization due to Aβ_25–35_ incorporation. The DPPC/Aβ_25–35_ and DMPC/Aβ_25–35_ membranes undergo an initial transition from EULVs (outer diameter of 800 Å) to BLSs (outer diameter of 400 Å), and further transition reversibly between the BLSs and SULVs (outer diameter of 200 Å) during their phase transitions. Our observations are consistent with membrane damage and its subsequent reformation being caused by the separate peptides or their small oligomers.

## Material and methods

### Sample preparation

1,2-Dimyristoyl-*sn*-glycero-3-phosphocholine (DMPC), 1,2-dipalmitoyl-*sn*-glycero-3-phosphocholine (DPPC) from Avanti Polar Lipids (Alabaster, AL) and amyloid-beta peptide segment 25–35 (Aβ_25–35_) from Abbiotec (Escondido, CA) were purchased in lyophilized powder form and used without further purification. Organic solvents 2,2,2-trifluoroethanol (TFE), chloroform, and trifluoroacetic acid (TFA) were purchased from Sigma-Aldrich Chemie Gmbh (Schnelldorf, Germany).

The lipid was dissolved in an organic solvents mixture of chloroform:TFE = 1:1 (by volume) in glass vials at the total concentration of 50 mg/ml. The peptide was subjected to a pretreatment procedure, according to which it was dissolved in TFA and treated in the ultrasonic bath for 10–12 min to enforce a peptide disaggregation^33^. The acid solution was then evaporated under a stream of nitrogen and the peptide was redissolved in the same organic solvent as the lipid at a concentration of 1.6 mg/ml.

The lipid and Aβ_25–35_ solutions were mixed in microtubes at the 0.5 mol% concentration of peptide, which was selected to diminish the possibility of its spontaneous aggregation and bilayer structure disruption^17^. The mixtures were dried to dryness and placed under a vacuum for 12 h for removing the remaining solvent. The lipid/Aβ_25–35_ films were then hydrated with D_2_O at their total concentration of 1% (by mass). The hydrated samples were frozen and thawed in several cycles accompanied by thorough vortexing. The attention was paid to keeping the samples at room temperatures at all times during the preparation procedure (i.e., below the DPPC phase transition from its gel phase).

The resulting multilamellar solutions were finally extruded into unilamellar vesicles (ULVs). The Avanti Mini Extruder was utilized at room temperature without heating, and fitted with polycarbonate membranes of sequentially decreasing pore sizes: 2000 Å, 1000 Å, and 500 Å. Only the finally extruded ULVs were used in the measurements. Independent samples of all systems were prepared for several experimental cycles.

### Small angle neutron scattering experiments

SANS measurements were performed at the time-of-flight spectrometer YuMO at the IBR-2 pulsed reactor (JINR, Dubna, Russia)^34^. The beam of neutrons was formed by a set of two pinhole collimators of diameters 40 and 14 mm. The neutrons scattered by the sample were detected with two circular wire-ring detectors placed at distances 4.5 and 13 m from the sample. An absolute intensity calibration was performed with the vanadium standard during the raw data treatment in the SAS program^35^. The liquid samples were contained in 2-mm-thick flat quartz cuvettes (Hellma) and held in the multipositional sample holder connected to the Lauda liquid thermostat with temperature controller Pt-100 that allows for a temperature accuracy of ± 0.03 °C.

The different samples of both systems of interest were examined in several independent experimental cycles. The standardly accessible range of scattering vector q from 0.007 to 0.5 Å^−1^ was extended in one of the measurements down to 0.005 Å^−1^ utilizing a cold-moderator setup^36^. This low-q enhanced data allowed us to corroborate our conclusions regarding the overall shape of examined objects (see Fig. S1 of the supplement). The collected scattering curves were corrected for background scattering from the buffer solution. The analysis of final SANS curves including the error analysis was performed with the SASFit program^29^ using the models of bilayered vesicles or randomly oriented cylindrical core–shell objects with a circular cross-section (see Fig. S7 of the supplement for the detailed description of models employed).

### Transmission electron microscopy

DMPC (1 wt%) and DMPC/Aβ_25–35_ (1 wt% total, and 3 mol% of Aβ_25–35_) solutions were diluted in deionized water to a final concentration of 67 µg/ml. The samples were set on carbon-coated 200 mesh copper grids with a thin layer of carbon 10 nm. Grids were treated by glow discharge using a PELCO easiGlow glow discharge cleaning system for a total of 25 s at 15 mA. Samples were placed onto the grids immediately following the glow discharge. The drop of solution was applied for 2 min to the metal-coated face of the grid. Samples were stained with aqueous uranyl acetate (2%) by soaking in solution for 1 min. Then, the grid with the sample was washed in deionized water for 1 min and air dried.

The micrographs were recorded at a magnification of 10,000–40,000 in Tecnai Polara G2 (FEI) transmission electron microscope (FEG cathode source operated at 300 eV of accelerating voltage) with the Gatan Orius 4k × 2.67k digital camera. The specimen received a dose of electrons 10e/Å^2^ in each exposure.

### Molecular dynamics simulations

The behavior of Aβ_25–35_ peptide in a model phospholipid membrane and its effect on membrane properties was investigated also utilizing a set of MD simulations. The simulations were performed using the GROMACS 2019.3 (http://www.gromacs.org) software package^37^ and the all-atom CHARMM36m force field^38^. The initial configurations and topologies of the model bilayers containing 256 DPPC molecules, 8 Aβ_25–35_ molecules, and 50 water molecules per lipid were set up using Membrane Builder from the CHARMM-GUI^39^. The bonds with H-atoms were constrained using the LINCS algorithm, force-based switching functions with a range of 1.0–1.2 nm were used for the Lennard–Jones interactions, and long-range electrostatic interactions were calculated using the particle mesh Ewald algorithm. Periodic boundary conditions were used in all three dimensions. The integration of the motion equations was performed using the leapfrog algorithm with a time step of 2 fs. The systems were coupled to the Berendsen thermostat at 293 K, 303 K, 313 K, and 323 K with a coupling time constant of 1 ps, while the pressure was maintained at 1 bar using a semi-isotropic pressure coupling with the Berendsen barostat. For production runs, the thermostat and barostat were switched to those of Nose–Hoover and Parrinello–Rahman, respectively. Equilibration was performed for 50 ns and 100 ns for the pure bilayers and peptide containing bilayers, respectively. The production simulations were carried out for up to 200 ns for pure lipid membranes, and 1 μs for the Aβ_25–35_ containing systems. The last 100 ns and 500 ns, respectively, were used for system analysis. The bilayer thickness was calculated based on the distribution density of phosphorus atoms of the lipid head groups using the in-house tools.

## Supplementary Information

Supplementary Information.
