# Supplementary material for: Amyloid-beta peptide (25–35) triggers a reorganization of lipid membranes driven by temperature changes
Source: Sci Rep. 2021 Nov 9;11:21990. doi: 10.1038/s41598-021-01347-7 (PMC8578324; doi:10.1038/s41598-021-01347-7)
Supplement: Supplementary file 1 — Supplementary Information. [file 41598_2021_1347_MOESM1_ESM.pdf]

Supplementary Information

Amyloid-beta peptide (25-35) triggers a reorganization of lipid membranes driven by temperature changes

Oleksandr Ivankov[1,2,*] Tatiana N. Murugova[1], Elena V. Ermakova[1], Tomáš Kondela[1,3], Dina R. Badreeva[4], Pavol Hrubovčák[1,5], Dmitry Soloviov,[1,2,6] Alexey Tsarenko[6], Andrey Rogachev[1,6], Alexander I. Kuklin[1,6] & Norbert Kučerka[1,7,*]

[1] Frank Laboratory of Neutron Physics, Joint Institute for Nuclear Research, Dubna, 141980, Russia

[2] Institute for Safety Problems of Nuclear Power Plants NAS of Ukraine, Kyiv, 03028, Ukraine

[3] Department of Nuclear Physics and Biophysics, Comenius University in Bratislava, Bratislava, 842 48, Slovakia

[4] Laboratory of Information Technologies, Joint Institute for Nuclear Research, Dubna, 141980, Russia

[5] Department of Condensed Matter Physics, University of P. J. Šafárik in Košice, Košice, 041 54, Slovakia

[6] Moscow Institute of Physics and Technology, Dolgoprudny, 141701, Russia

[7] Department of Physical Chemistry of Drugs, Faculty of Pharmacy, Comenius University in Bratislava, Bratislava, SK-832 32, Slovakia

*E-mail: ivankov@jinr.ru, kucerka@nf.jinr.ru

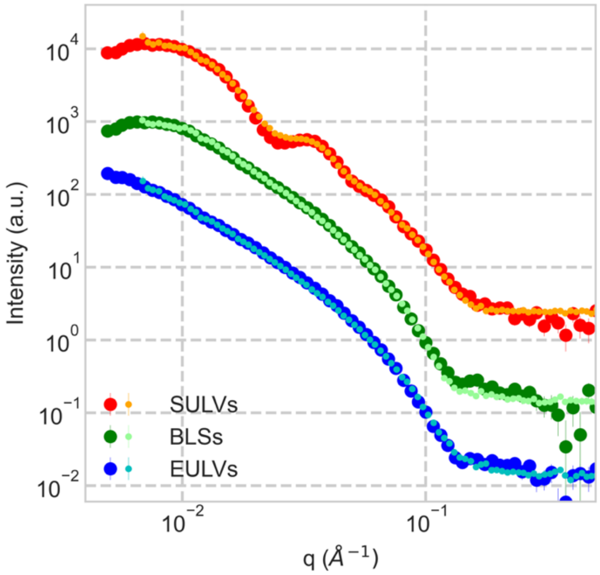

Figure S1. The low-q enhanced data (large dots) compared to the data of standard setup (small dots). The different forms of scattering curves allow distinguishing the overall shape of membrane organization (SULVs vs. BLSs vs. EULVs).

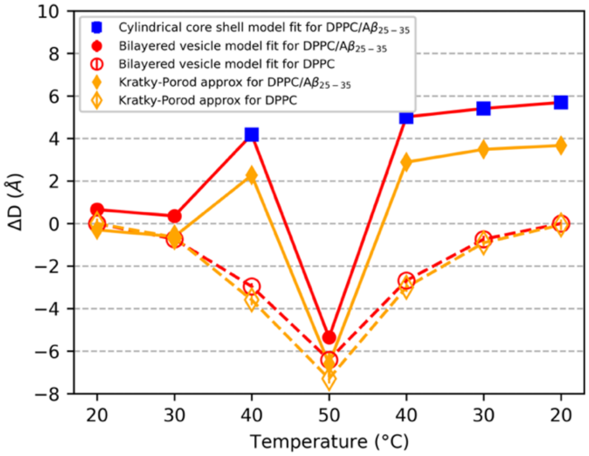

Figure S2. Comparison of the membrane thickness relative changes obtained by the Kratky-Porod approximation or by fitting the appropriate models (bilayered vesicles or randomly oriented cylindrical core-shell objects with a circular cross-section). The changes in the obtained membrane thicknesses are independent of the approximation model (compare the two broken lines) or the model describing an overall shape of the membrane (compare the two solid lines).

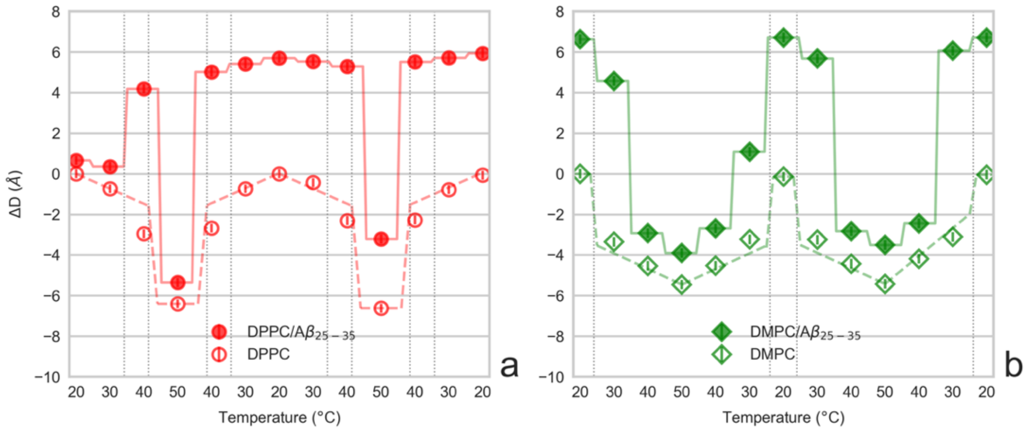

Figure S3. The relative changes to membrane thickness ΔD obtained for the systems based on DPPC (A) or DMPC (B). The neat lipid systems are shown by empty symbols and those with the addition of A$\beta_{25\text{-}35}$ by solid ones. The changes are shown with respect to the neat lipid bilayer thickness obtained at T=20°C. The vertical lines demarcate the phase transition temperatures for neat lipid systems. The solid lines were added to guide the eyes, while the dashed lines were adopted from the investigations of neat lipid bilayers [1].

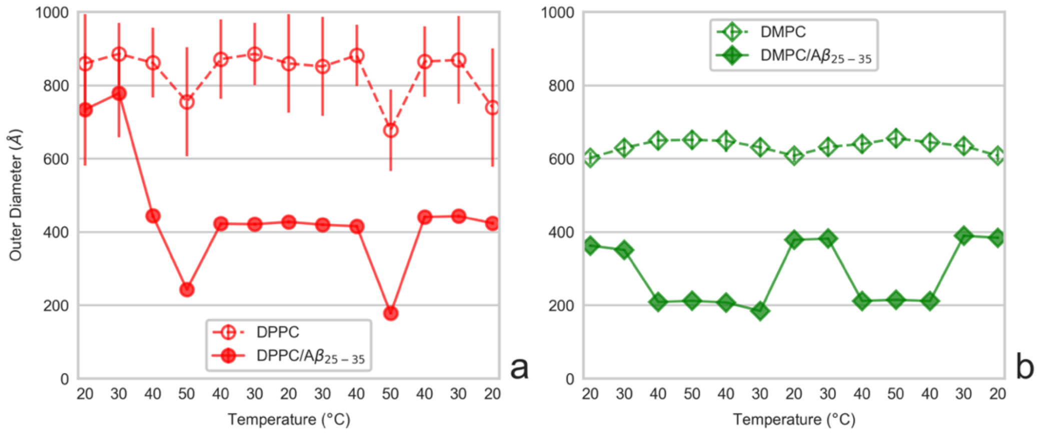

Figure S4. The outer diameters of spherical ULVs or the circular cross-section outer diameters of BLSs where appropriate, as obtained from SANS data for the systems based on DPPC (A) or DMPC (B). The neat lipid systems are shown by empty symbols and those with the addition of A$\beta_{25\text{-}35}$ by solid ones. The elevated errors are obtained for the systems with a high-level polydispersity.

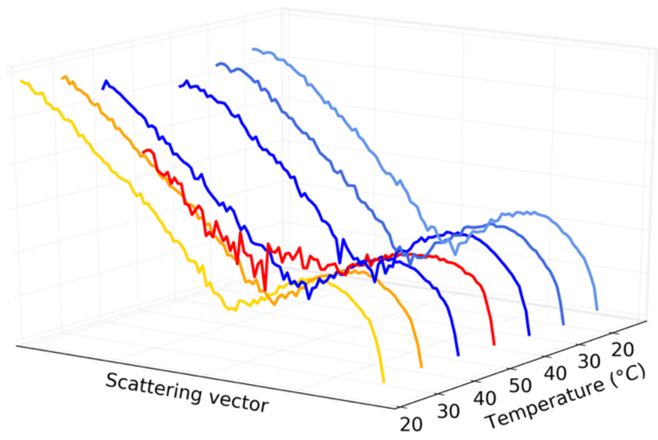

Figure S5. The complementary SAXS measurements for DPPC/A$\beta_{25\text{-}35}$ system at various temperatures. The curves corroborate the conclusions based on SANS data.

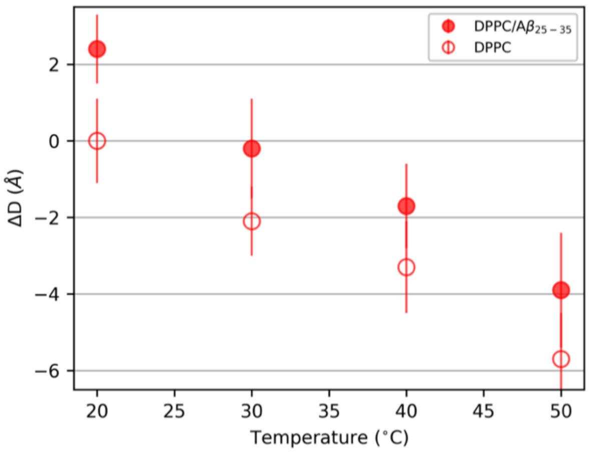

Figure S6. The relative changes to membrane thickness ΔD obtained from MD simulation results of DPPC (empty circles) and DPPC/A$\beta_{25\text{-}35}$ (solid circles) systems. The relative changes are shown with respect to the neat DPPC bilayer thickness obtained at T=20°C.

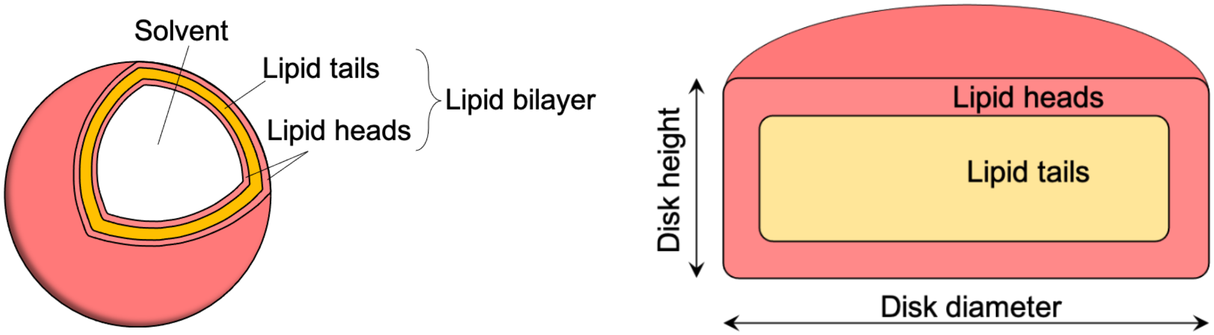

Figure S7. The analysis of experimental SANS curves was performed with the SASFit program [2] using the models of bilayered vesicles (left) or randomly oriented cylindrical core-shell objects with a circular cross-section (right). The scattering intensity was calculated in the case of the bilayered vesicle model as

$$I_{BLV}(Q) = \left(K(Q,R_c,\eta_{sol}-\eta_t) + K(Q,R_c+t_t,\eta_t-\eta_h) + K(Q,R_c+t_t+t_h,\eta_h-\eta_t) + K(Q,R_c+2t_t+t_h,\eta_t-\eta_{sol})\right)^2$$

with $K(Q,R,\Delta\eta) = \frac{4}{3}\pi R^3 \Delta\eta 3 \frac{\sin(QR) - QR\cos(QR)}{(QR)^3}$,

where R_c is a radius of a core consisting of solvent, t_h is the thickness of lipid heads region and t_t that of lipid tails (from both monolayers), and $\eta_{sol}, \eta_h, \eta_t$ are the scattering length densities of solvent, lipid heads, and lipid tails, respectively. The intensity in the case of the cylindrical shell model was calculated as

$$I_{CylShell} = \int_0^1 \left(K_{Cyl}(Q,\eta_{core}-\eta_{shell},R,L,x) + K_{Cyl}(Q,\eta_{shell}-\eta_{solv},R+t,L+2t,x)\right)^2 dx$$

with $K_{Cyl}(Q,\Delta\eta,R,L,x) = 2\pi R^2 L \Delta\eta \frac{J_1(QR\sqrt{1-x^2})}{QR\sqrt{1-x^2}} \frac{\sin(QLx/2)}{QLx/2}$,

where R, L are the radius and height of core consisting of lipid tails, a is the thickness of shell (lipid heads), and a are the scattering length densities of solvent, shell (lipid heads), and core (lipid tails), respectively.

References

1 Kuklin, A. *et al.* On the Origin of the Anomalous Behavior of Lipid Membrane Properties in the Vicinity of the Chain-Melting Phase Transition. *Scientific Reports* **10**, 5749, doi:10.1038/s41598-020-62577-9 (2020).

2 Breßler, I., Kohlbrecher, J. & Thünemann, A. F. SASfit: a tool for small-angle scattering data analysis using a library of analytical expressions. *Journal of Applied Crystallography* **48**, 1587-1598, doi:10.1107/S1600576715016544 (2015).
